# Implantable Defibrillator in the Systemic Right Ventricle: Are We Still Baffled?

**DOI:** 10.1016/j.cjcpc.2025.05.001

**Published:** 2025-05-22

**Authors:** Sakethram Saravu Vijayashankar, Shubhayan Sanatani

**Affiliations:** Division of Cardiology, Department of Pediatrics, University of British Columbia, Vancouver, British Columbia, Canada

The prevalence of congenital heart disease (CHD) has remained relatively stable in newborns over the past several decades. With improvements in cardiac management, there are now more adults with CHD (ACHD) than children in most of the developed world.^1^ A unique challenge that remains is the management of a patient with a systemic right ventricle (sRV). Two prevalent conditions are D-transposition of the great arteries (D-TGA) repaired with an atrial baffle procedure and congenitally corrected transposition of the great arteries (CCTGA). Dr Senning performed the first atrial switch in 1957; Dr Mustard provided a modification of this procedure in 1963, the now eponymous Mustard procedure. The atrial switch was the only surgical repair for the management of D-TGA until Dr Jatene described the first successful arterial switch procedure in 1975. Despite the appeal of restoring a systemic left ventricle, the atrial switch continued to be performed until the late 1980s and beyond, as institutions gradually transitioned to the arterial switch. The atrial switch is still indicated in some uncommon presentations. Survival of neonates with transposition has been a tremendous success of the paediatric CHD community. A Congenital Heart Surgeons Society study from 2003 reported that 94% of patients who underwent an atrial switch were alive at 17 years of follow-up, vs 81% for arterial switch patients.^2^ This reflects the early era of the arterial switch and the challenges in mastery of the new management strategies. The predominant strategy for TGA for decades, with excellent survival rates, the ACHD community now cares for a large cohort of patients who underwent the atrial switch procedure.^3^

The functional status of the sRV however revealed a different story. The arterial switches were faring much better than their atrial switch counterparts as the years passed.^4^ It became clear that there was a slow decline in ventricular function late after repair and that the process was progressive. Patients with CCTGA similarly suffer a progressive decline in cardiac function with time. A large multicentre study showed that 25% of patients with CCTGA required mechanical circulatory support and transplant, or were deceased by 50 years of age.^5^ Atrial arrhythmias, β-blocker use, implantable cardioverter defibrillators (ICDs), diabetes, and obesity were noted to be significantly associated with these poor outcomes. This showed that the natural progression of cardiac dysfunction was marred by electrical instability and the added burden of acquiring adult-onset degenerative diseases.^5^

Sudden cardiac death (SCD) continues to be a major concern in the sRV, accounting for rates up to 25% per 1000 patient-years.^6^ SCD is not always a singular diagnosis and is often a culmination of multiple factors over the years. Ventricular arrhythmias are often implicated in SCD, especially in patients with an atrial switch procedure and the sRV. However, the effects of atrial arrhythmias, systemic effects of heart failure, and lifelong consequences of the sRV supporting systemic circulation all contribute.^6^ Detection of spontaneously terminating ventricular arrhythmias in ICD tracings shows us that ventricular rhythms are likely overestimated as the sole causative factor in this cohort.^7^

Traditional predictors of SCD noted in the systemic left ventricle have not been directly applicable to the sRV population, and knowledge is lacking regarding predictors of SCD in sRV. The benefits of ICDs in this population are difficult to gauge given the frequency of inappropriate shocks and lead complications, the younger age of patients, and the high prevalence of atrial arrhythmias. The natural history of arrhythmias in the sRV appears to be progressive with time. van Dissel et al.^5^ showed that there was a natural progression in electrical instability with the initial detection of complete heart block requiring pacemaker implantation, followed by atrial and finally ventricular arrhythmias in the CCTGA cohort over time. A study by Nozica et al.^8^ showed that 25% of patients with an atrial switch procedure had a pacemaker or ICD implanted by a median age of 22 years. Half of the patients in the cohort had experienced a clinically significant arrhythmia. Death, circulatory support, or transplant was noted in 10% of these patients, and a significant number (9%) experienced SCD or nonfatal ventricular arrhythmias. Thus, there is a strong triad of heart failure, ventricular dysfunction, and arrhythmias noted in patients with an sRV over time. To delve into these questions, Yao et al.^9^ present the results of a systematic review of ICD therapy in the sRV.

The authors' primary aim was to tease out patient factors that might predict appropriate ICD discharge in the sRV. They performed a meta-analysis of available studies to draw their conclusions. The search strategy identified 40 papers, of which 11 were included in the final analysis. The authors used established methods for the review and graded the quality of the papers selected. The papers were published from 2008 until 2022 from Europe, North America, and Australia. Most of the included papers consisted of a small cohort of patients, with most of the subjects representing just over 40% of all the ICDs studied coming from one paper.^10^ This shows the difficulty that exists in gathering data in this niche cohort. They examined rates of inappropriate discharges and complications associated with ICD implantation. They divided their results as primary and secondary prevention to see if further clues can be gained regarding selection criteria that may help in the future. The oldest patient in this systematic review was born in 1962, thus representing the various eras of the atrial switch procedure in their cohort.

The authors found that 9.1% of patients with primary prevention ICDs and 34.9% of secondary prevention ICDs received appropriate therapies during a mean or median follow-up time, ranging from 19 months to 9.4 years. The ratio of appropriate to inappropriate shocks in the primary prevention group was 0.5:1, compared with 3:1 in the secondary prevention group. Three-quarters (76%) of inappropriate shocks were due to atrial arrhythmias, and 22% were due to lead malfunction. Using a Poisson model, it was found that total appropriate shocks occurred at a marginally higher rate, which was not statistically significant. Lead complications were seen in up to 8.4% of patients. Most common clinical reasons for ICD implant included sRV dysfunction and sustained and nonsustained ventricular tachycardia on Holter monitoring, exercise testing, and electrophysiology study.

Indications for ICD placement in the sRV continue to represent a knowledge gap in the ACHD population. The indications for primary prevention ICD placement in this population remain murky. The Pediatric and Congenital Electrophysiology Society guidelines^11^ continue to be an important document for guiding the management of patients with sRVs at risk of SCD. The prevention-ACHD risk score added comorbidities acquired through life as an important determinant in calculating the risk of SCD in patients with not only an sRV but various other ACHD pathologies.^12^ These have helped the decision-making matrix, but the patient with an implanted ICD continues to be burdened with high rates of inappropriate shock and lead complications.

The authors have done a good job extracting the data from a diverse literature, with different methodologies and amounts of patient-level detail. The type of ICD was not available in nearly half of the included patients. The absence of control data is another limitation of this paper. Most of the studies did not perform a risk factor analysis; thus, a formal meta-regression could not be performed. No comparison was made between CCTGA and sRV either. This is likely an important contributor as demonstrated by Ladouceur et al. in 2022.^10^ A major factor that was not studied in this paper is the process of aging and its various comorbidities such as coronary artery disease or even patient symptomatology. These are likely important factors to consider in these patients as they continue to acquire various maladies with greater age. Understandably, the study did not determine what changes in mortality/morbidity was seen in patients after the implantation of the ICD. The limitations have been well highlighted by the authors. They propose further studies. Perhaps a future direction would be to investigate quality of life in patients with and without an ICD.

In summary, we commend the authors who have shed light on this topic, which continues to challenge electrophysiologists caring for patients with ACHD. The systematic review did not show a statistical significance between appropriate and inappropriate shocks in the primary prevention cohort. They thus reinforce that considerate decision-making is required while placing ICDs in the sRV. They have shown that traditional indications and guidelines for primary prevention are unsatisfactory, with inappropriate shocks and complications occurring at high frequencies. There is a need for robust validated scores to optimize the selection of sRV patients for ICD implants to reduce inappropriate shocks and complications in this vulnerable cohort. Aggressive management of atrial arrhythmias is warranted to prevent inappropriate shocks. The number of sRV patients is likely to diminish over time, given the current management of TGA. Our window of opportunity to understand risk prediction in this condition may be limited. In the meantime, Yao et al. have given us an excellent collation of data to consider in our discussions with patients.
